# Supplementary material for: The role of deliberate practice in expert performance: revisiting Ericsson, Krampe & Tesch-Römer (1993)
Source: R Soc Open Sci. 2019 Aug 21;6(8):190327. doi: 10.1098/rsos.190327 (PMC6731745; doi:10.1098/rsos.190327)
Supplement: Additional Methods and Results [file rsos190327supp1.docx]

**Additional Methods**

**The Professional Violinists**

**Original study methods.** According to the music academy professors in Ericsson et al. ^[S1]^, the best violinists were likely to have careers as members of a top German orchestra. For this reason, Ericsson et al. additionally recruited 10 male professional violinists (mean age 50.5) from two orchestras in West Berlin with international reputations (the Berlin Philharmonic Orchestra and the Radio Symphony Orchestra) to compare with the best violinists. Ericsson et al. collected data from them using a modified procedure (only Session 1). The professional violinists were not included in any of the inferential analyses, with the exception of demographics and a comparison of accumulated practice alone until age 18 with the best violinists where no differences were observed. Likewise, the professionals' descriptive data were only provided for demographics and retrospective practice histories (via figures).

**Replication study methods.** We were able to recruit four professional violinists (3 females; ranging in age from 27 to 54 at the time of the interview) from the Cleveland Orchestra. The Cleveland Orchestra is one of "the big 5" major American orchestras and is ranked 7^th^ in the world ^[S2]^. Following Ericsson et al., we used a modified procedure with the professional violinists (the professional violinists only participated in Session 1). We therefore could not implement a double-blind procedure with this group. They additionally responded to the awareness and beliefs questionnaire (described below). Due to the small size, we do not conduct any inferential statistics with this group. Rather, we report relevant descriptive statistics.

**Replication Questionnaires**

**Diary debriefing and questionnaire*.***

***Original study methods.*** Ericsson et al. asked participants to fill out a questionnaire asking about the diary process and the week, such as if the diary week was representative of a typical week.

***Replication study methods.*** As in Ericsson et al., participants filled out a questionnaire asking about the diary process and the week, such as if the diary week was representative of a typical week.

**Practice intensity and goals in practice*.***

***Original study methods.*** Participants completed a questionnaire on their concentration and practice goals

***Replication study methods.*** As in Ericsson et al., participants completed a questionnaire on their concentration and practice goals as in Ericsson et al.

**Additional Measures Included Following all Replication Measures**

**Questionnaires.**

***Mindset ^[S3]^.*** Participants responded to eight statements about intelligence and eight statements about talent (e.g., "Your intelligence/talent is something about you that you can't change very much.") "Strongly Agree," "Agree," "Mostly Agree," "Mostly Disagree," "Disagree," or "Strongly Disagree." Responses were coded as 1, 2, 3, 4, 5, or 6 respectively and reverse scored when appropriate such that higher scores reflected more of a growth mindset—believing intelligence and/or talent can change with effort—and lower scores reflected more of a fixed mindset—believing intelligence and/or talent are relatively stable.

***Awareness and beliefs questionnaire.*** We designed a questionnaire to gauge participants' familiarity with and beliefs about predictors of expertise. The questionnaire consisted of 4 items.

The first item asked participants, "Have you ever heard of the "10,000 hour rule?" If participants marked "yes," they were asked to indicate where they heard about it. The options were "read Malcolm Gladwell's book 'Outliers,'" "read about it in a magazine article/newspaper article/other book." "read about it online," or "heard about it from a friend, colleague, family, or other." If participants indicated the last option, they were asked to indicate which of those sources best described where they heard it. The second item asked participants, "Have you heard of "deliberate practice?" The third item asked participants, "Do you believe that with 10,000 hours of practice anyone can become an expert at anything?" The fourth item gave lay definitions of 'nature:' "(includes talent, innate ability, personality, and genes)" and 'nurture:' "(includes practice, training, encouragement, and opportunity)." Following these definitions, participants indicated the proportion (out of 100%) that they believe "nature" and "nurture" account for their own performance level on the violin. Next, we asked participants to indicate these proportions for their peers' performance levels on the violin.

**Cognitive ability measures.**

***Working memory capacity.***  We included three measures of working memory capacity. The mean proportion correct on the three measures serves as the composite working memory capacity score.

*Operation Span.* In the shortened operation span task ^[S4]^ participants solve simple math problems while attempting to remember letters. After 4-6 math problem-letter pairs, participants are asked to recall the letters in serial order. The shortened Operation Span task takes approximately 20 minutes.

*Symmetry Span.* In the shortened Symmetry Span task ^[S5]^ participants judge whether an image is symmetrical along the vertical axis while attempting to remember the location of filled squares in a grid. After 3-5 symmetry problem-square location pairs, participants are asked to recall the square locations in serial order. The shortened Symmetry Span task takes approximately 17 minutes.

*Letter-Number Sequencing.* In the letter-number sequencing task ^[S6]^ participants mentally reorder a series of digits and letters. After a list of 3-7 items (a combination of digits and letters, e.g., T, 8, D, 3) are presented one at a time, participants are asked to recall first the digits in ascending order followed by the letters in alphabetical order. For example, if participants were presented with the sequence T, 8, D, 3, the correct response is 3, 8, D, T. The letter-number sequencing task takes approximately 8 minutes.

***Fluid intelligence.*** We included two measures of fluid intelligence: Raven’s Advanced Progressive Matrices ^[S7]^ and Letter Sets ^[S8]^. The mean proportion correct on the two measures serves as the composite fluid intelligence score. A number of other intelligence measures could have been included, such as the Wonderlic test ^[S9]^. We did not include the Wonderlic because evidence for its validity is limited both in terms of being a consistent predictor of job performance and in terms of correlations with fluid intelligence (see, e.g., ^[S10]^).

*Raven's Advanced Progressive Matrices.* In Raven's Progressive Matrices ^[S7]^ (odd problems) participants view a 3 $\times$ 3 matrix with the lower right corner missing and are tasked with choosing the missing piece from a set of options that completes the pattern. After practice and instructions, participants are given 10 minutes to complete as many problems as they can.

*Letter Sets.* In Letter Sets ^[S8]^, participants view five letter strings per set and determine which letter string does not follow the rule for that set. After instructions and practice, participants are given 7.5 minutes to complete as many problems as they can.

***Cognitive processing speed.*** We include two measures of cognitive processing speed. The mean proportion correct on the two measures serves as the composite processing speed score.

*Letter Comparison.* In Letter Comparison ^[S11]^, participants determine as quickly as they can whether pairs of letter strings are the same or different. After instructions and practice, participants are given 30 seconds per page to complete as many items as they can.

*Pattern Comparison.* In Pattern Comparison ^[S11]^, participants determine as quickly as they can whether pairs of figures are the same or different. After instructions and practice, participants are given 30 seconds per page to complete as many items as they can.

***Basic music ability.*** Basic music ability is measured using the Swedish Music Discrimination Task ^[S12]^, which is composed of three sub-tests: basic pitch discrimination, basic rhythm discrimination, and basic melody discrimination. Performance on these tasks has been shown to be highly genetic, with no influence of deliberate practice on performance after controlling for heritability. The mean proportion correct on the three sub-tests serves as the composite basic music ability score. Participants listened to the stimuli using Sennheiser HD 280 Professional headphones.

*Basic pitch discrimination.* In this sub-task participants indicate whether the second tone in a pair is higher or lower than the first tone in the pair. This sub-task takes approximately 3 minutes.

*Basic rhythm discrimination.* In this sub-task participants indicate whether pairs of rhythmic sequences are the same or different. This sub-task takes approximately 3 minutes.

*Basic melody discrimination.* In this sub-task participants indicate which tone in pairs of tone sequences differs between the sequences. This sub-task takes approximately 5 minutes.

**Additional Measures Procedure**

Additional measures were administered following all other replication measures, but prior to the debriefing. The order of these measures were as follows: basic rhythm ability, Raven's Advanced Progressive Matrices, Symmetry Span, Letter Comparison, Letter-Number Sequencing, mindset questionnaire, basic tone ability, Letter Sets, Operation Span, Pattern Comparison, basic pitch ability, and the awareness and beliefs questionnaire. Measures were administered to all student violinists for blinding purposes. However, only the cognitive ability scores of the best and good violinists who had the same college admission criteria were examined.

**Additional Results**

In cases where assumptions are not met, we use non-parametric tests: Kruskal-Walis tests in place of between-subject ANOVAs, Greenhouse-Geisser corrections for repeated measures ANOVAs, and Welch's tests in place of *t*-tests.

**Professional Violinists' Biographical Data**

**Original study results.** Ericsson et al. report the grand means (collapsed across student and professional participants) for age they began practicing, age they began lessons, age they first decided to become musicians, average number of music teachers, and the average number of musical instruments studied beyond the violin. The note that the groups did not differ from each other (statistics not reported). The professional violinists are not included in any further inferential tests with the exception that Ericsson et al. report that the professionals’ accumulated hours of practice alone up to age 18 did not differ significantly from the best violinists’ accumulated hours up to age 18 (statistical results not reported). Ericsson et al. ^[1]^ include the professional violinists’ data in two of the study’s eight graphs: estimated weekly practice alone by age and estimated accumulated practice to age 20.

**Replication study results.** See Table S1 for raw and descriptive statistics of the professionals’ violin playing musical histories: age they began practicing, age they began lessons, age they first decided to become musicians, average number of music teachers, and the average number of musical instruments studied beyond the violin.

| Table S1  *General Musical Histories of the Professional Violinists* | | | | | | | | | | |
| --- | --- | --- | --- | --- | --- | --- | --- | --- | --- | --- |
|  |  | *M* |  | P1 |  | P2 |  | P3 |  | P4 |
| Age began practicing the violin |  | 5.00 |  | 6 |  | 5 |  | 6 |  | 3 |
| Age began violin lessons |  | 5.00 |  | 6 |  | 5 |  | 6 |  | 3 |
| Age decided to pursue music as a career |  | 17.25 |  | 14 |  | 18 |  | 19 |  | 18 |
| Number of violin teachers |  | 7.25 |  | 7 |  | 5 |  | 4 |  | 13 |
| Number of other instruments played |  | 1.25 |  | 0 |  | 1 |  | 3 |  | 1 |
| Years practicing the violin |  | 34.75 |  | 40 |  | 22 |  | 48 |  | 29 |
| Note. P1 = Professional 1, P2 = Professional 2, P3 = Professional 3, P4 = Professional 4 | | | | | | | | | | |

In examining the retrospective practice histories of the older professional violinists in the present study, we noticed a marked shift in amount of weekly practice around age 20. Specifically, the increasing monotonic pattern until around age 20 (see Figure S1, left panel) shifts, such that after age 20, variability increases and the overall number of weekly amounts of practiced alone per week decreases (see Figure S1, right panel).

*Figure S1.* Estimated amounts of weekly practice alone with the violin among the professional violinists as a function of age.

The shift around age 20 is even more striking when examining teacher-designed practice. Again, like Ericsson et al., we observe a monotonic increase in amount of practice per week to age 20 (see Figure S2, left panel). However, after age 20, the amount of teacher-designed practice declines rapidly (see Figure S2, right panel). In each case, once the violinist reported being hired by a professional orchestra, the violinist stopped engaging in teacher-designed practice altogether. This ceasing of all teacher-designed practice is note-worthy because if deliberate practice is defined as teacher-designed practice, then one must accept that at least some of the highest-level professional musicians do not engage in deliberate practice.

*Figure S2*. Hours per week of teacher-designed practice with the violin as a function of age among the professional violinists.

Consistent with Ericsson et al.'s (1993) findings, the average accumulated amounts of practice alone for the best violinists and the professional violinists were nearly identical throughout their development, see Figure S3.

*Figure S3.* Accumulated amount of practice alone as a function of age for the best (student) violinists and the professional violinists. Error bars represent 95% confidence intervals.

The average accumulated amounts of teacher-designed practice of the professional violinists was also very similar to that of the best violinists, see Figure S4.

*Figure S4.* Accumulated amount of teacher-designed practice as a function of age for the best (student) violinists and the professional violinists. Error bars represent 95% confidence intervals.

**Additional Student Violinist Results**

We lost one student violinist from the best group to attrition. For measures following Session 1 our *N* = 38.

**Music Memorized**

**Original study results.** Ericsson et al. (1993) asked the student violinists to estimate how much music in minutes they could perform from memory without preparation. They reported two ANOVAs, one comparing the best violinists’ mean memorized minutes of music to the good violinists’ mean memorized minutes of music (excluding the less accomplished violinists but using the full sample degrees of freedom), and the other ANOVA combining the best and good violinists into a single group and comparing their combined mean memorized minutes of music to the less accomplished violinists’ mean memorized minutes of music. Both analyses were significant. Ericsson et al. found that the best violinists' average (128.9 minutes) was significantly more than the good violinists' average (79.1 minutes). They also reported that the average minutes of music memorized by the best and good violinists combined (104.0) was significantly more than the average for the less accomplished violinists (42.27).

**Replication study results.** The violinists in the present study estimated substantially more minutes of music on average they could perform from memory without preparation than the violinists in Ericsson et al. (1993). In the present study, variability was high and the differences across groups were not significant, χ^2^(2) = 1.16, *p* = .559, η^2^ = .06. The best violinists reported an average of 7,223.35 minutes (95% CI [0.00^[[1]](#footnote-1)^, 20,904.16], range = 30–84,000), which was not significantly greater than the good violinists' average of 448.85 (95% CI [0.00^1^, 1,065.64], range = 40–4,200), *t*(11.05) = 0.97, *p* = .353, *d* = 0.42. The average of the good violinists was not significantly greater than the less accomplished violinists' average of 130.00 (95% CI [60.52, 199.48], range = 20–420), *t*(24) = 1.01, *p* = .324, *d* = .41 . One outlier was observed. One of the best violinists reported having almost 34 times as many minutes memorized as the grand mean. When replacing this outlier with the group mean, we still do not observe a main effect of group, *F*(2, 35) = 0.87, *p* = .429, η^2^ = .05. The best violinists (*M* = 825.30, 95% CI [0.00^1^, 1927.53], range = 30–7223.35) had not memorized significantly more minutes of music than the good violinists, *t*(23) = 0.58, *p* = .568, *d* = 0.24.

**Ratings of Musical and Everyday Activities by Group**

**Original study results.** Ericsson et al. report that there were no differences in how the student violinists rated the activities and that there was no interaction between group and ratings (statistical results not reported).

**Replication study results.** We tested whether any of the activity ratings differed by group, correcting for multiple comparisons. See Table S5 for tests of ratings varying by group. Like Ericsson et al.'s findings, there were no significant group difference in activity ratings. We also tested whether there was a significant interaction between group and rating type. Our three Greenhouse-Geisser corrected (because Mauchley's test was significant) 3 (skill level) × 25 (activity) ANOVAs also revealed that the interactions, like those in Ericsson et al. (1993), were not significant. Relevance: *F*(18.69, 327.06) = 1.54, *p* = .072; Effort: *F*(24.54, 429.45) = 1.12, *p* = .311; Enjoyment: *F*(24.25, 424.29) = 1.22, *p* = .217.

| Table S5  *Tests of Student Group Differences By Activity Rating* | | | | | | |
| --- | --- | --- | --- | --- | --- | --- |
|  |  | Musical Activities | | | | |
|  |  | Relevance |  | Effort |  | Enjoyment |
| Practice alone |  | χ^2^_(2)_=1.03, *p*=.598 |  | *F*_(2, 36)_=0.72, *p*=.495 |  | *F*_(2, 36)_=3.77, *p*=.033 |
| Practice activities  designed by teacher |  | χ^2^_(2)_=3.58, *p*=.167 |  | *F*_(2, 36)_=0.49, *p*=.615 |  | *F*_(2, 36)_=1.10, *p*=.345 |
| Practice with others |  | *F*_(2, 36)_=0.47, *p*=.632 |  | *F*_(2, 36)_=0.76, *p*=.475 |  | *F*_(2, 36)_=0.98, *p*=.385 |
| Playing for fun alone |  | *F*_(2, 36)_=1.07, *p*=.354 |  | *F*_(2, 36)_=1.44, *p*=.250 |  | *F*_(2, 36)_=1.61, *p*=.214 |
| Playing for fun with  others |  | *F*_(2, 36)_=0.60, *p*=.553 |  | *F*_(2, 36)_=0.38, *p*=.685 |  | *F*_(2, 36)_=0.11, *p*=.901 |
| Taking lessons |  | χ^2^_(2)_=4.82, *p*=.090 |  | *F*_(2, 36)_=0.06, *p*=.940 |  | *F*_(2, 36)_=2.06, *p*=.143 |
| Giving lessons |  | *F*_(2, 36)_=1.00, *p*=.380 |  | *F*_(2, 36)_=0.13, *p*=.878 |  | *F*_(2, 36)_=1.48, *p*=.242 |
| Solo performance |  | χ^2^_(2)_=5.84, *p*=.054 |  | χ^2^_(2)_=1.05, *p*=.592 |  | *F*_(2, 36)_=0.01, *p*=.987 |
| Group performance |  | *F*_(2, 36)_=2.25, *p*=.120 |  | *F*_(2, 36)_=0.45, *p*=.642 |  | *F*_(2, 36)_=0.20, *p*=.820 |
| Listening to music |  | *F*_(2, 36)_=0.39, *p*=.679 |  | *F*_(2, 36)_=1.55, *p*=.227 |  | *F*_(2, 36)_=2.69, *p*=.081 |
| Music theory |  | *F*_(2, 36)_=3.36, *p*=.046 |  | *F*_(2, 36)_=1.91, *p*=.163 |  | *F*_(2, 36)_=3.25, *p*=.051 |
| Professional  conversation |  | *F*_(2, 36)_=3.80, *p*=.032 |  | *F*_(2, 36)_=0.11, *p*=.898 |  | *F*_(2, 36)_=0.92, *p*=.408 |
| Organization and  preparation |  | *F*_(2, 36)_=1.07, *p*=.355 |  | *F*_(2, 36)_=0.24, *p*=.785 |  | *F*_(2, 36)_=0.44, *p*=.645 |
|  |  | Everyday Activities | | | | |
|  |  | Relevance |  | Effort |  | Enjoyment |
| Household chores |  | *F*_(2, 36)_=2.71, *p*=.080 |  | *F*_(2, 36)_=0.73, *p*=.490 |  | *F*_(2, 36)_=1.40, *p*=.261 |
| Child care |  | χ^2^_(2)_=4.81, *p*=.090 |  | *F*_(2, 36)_=0.46, *p*=.634 |  | *F*_(2, 36)_=2.50, *p*=.096 |
| Shopping |  | χ^2^_(2)_=5.38, *p*=.068 |  | *F*_(2, 36)_=1.19, *p*=.317 |  | *F*_(2, 36)_=1.67, *p*=.203 |
| Work (not music  related) |  | *F*_(2, 36)_=0.88, *p*=.423 |  | *F*_(2, 36)_=2.58, *p*=.089 |  | *F*_(2, 36)_=0.23, *p*=.797 |
| Sports/Fitness |  | *F*_(2, 36)_=1.22, *p*=.308 |  | *F*_(2, 36)_=1.40, *p*=.261 |  | *F*_(2, 36)_=0.22, *p*=.806 |
| Personal care |  | *F*_(2, 35)_=2.09, *p*=.139 |  | *F*_(2, 35)_=0.62, *p*=.543 |  | *F*_(2, 35)_=1.19, *p*=.315 |
| Sleep |  | *F*_(2, 36)_=2.70, *p*=.081 |  | *F*_(2, 36)_=0.10, *p*=.905 |  | *F*_(2, 36)_=1.33, *p*=.278 |
| Education (not music) |  | *F*_(2, 36)_=3.24, *p*=.051 |  | *F*_(2, 36)_=3.33, *p*=.047 |  | *F*_(2, 36)_=0.14, *p*=.870 |
| Committee work |  | *F*_(2, 36)_=0.74, *p*=.486 |  | *F*_(2, 36)_=1.40, *p*=.261 |  | *F*_(2, 36)_=2.58, *p*=.089 |
| Social activities |  | *F*_(2, 36)_=1.87, *p*=.169 |  | *F*_(2, 36)_=2.28, *p*=.116 |  | *F*_(2, 36)_=0.56, *p*=.577 |
| Social media/email |  | *F*_(2, 36)_=2.48, *p*=.098 |  | *F*_(2, 36)_=1.38, *p*=.264 |  | *F*_(2, 36)_=0.60, *p*=.553 |
| Leisure/hobbies^*^ |  | *F*_(2, 36)_=0.83, *p*=.444 |  | *F*_(2, 36)_=0.39, *p*=.681 |  | *F*_(2, 36)_=1.89, *p*=.166 |
| Note. Ratings not following a normal distribution were submitted to Kruskal-Wallis non-parametric tests. Bonferroni's corrected alpha = .0007. No group differences on any activity rating emerge using this criterion. Ratings for personal care are missing for one participant. | | | | | | |

**Diaries**

**Original study.** Ericsson et al. asked the student violinists to keep a diary log for seven days, logging and coding all activities according to the taxonomy of activities presented in Session 1. Participants were encouraged to identify the primary code for each activity though they were allowed to use two codes, such as having a professional discussion during lunch. In those cases, the time was split equally between the two activities. Ericsson et al. also asked participants to indicate whether the diary week was typical, however, they do not report the number who indicated the week was atypical. According to the degrees of freedom, all diaries were included in analyses.

**Replication study.** We asked the student violinists to keep a diary log for seven days, logging and coding all activities according to the taxonomy of activities presented in Session 1. Participants were encouraged to identify the primary code for each activity though they were allowed to use two codes, such as having a professional discussion during lunch. In those cases, the time was split equally between the two activities. We also asked participants to indicate whether the diary week was typical and analyzed results with and without those who indicated that the diary week was atypical.

**All musical activities.**

***Original study results.*** Ericsson et al.^[S1]^ analyzed time spent on all musical activities during the diary week. They found that the average amount of time spent on all music related activities summed across the diary week was 50.6 hours, with no differences between groups (statistical results not reported).

***Replication study results.*** Following Ericsson et al., we analyzed time spent on all musical activities during the diary week. The average amount of time spent on all music activities during the diary week for all student violinists was 44.82 hours (95% CI [38.69, 50.95], range = 7.88–83.50). We found a significant difference across groups, *F*(2, 35) = 7.40, *p* = .002, η^2^ = .30. Post-hoc follow-up tests revealed that the best violinists (*M* = 54.44, 95% CI [46.03, 62.84], range = 30.00–83.50) and the good violinists (*M* = 50.13, 95% CI [39.61, 60.64], range = 7.88–71.75) spent similar amounts of time engaged in musical activities, *p* = .795, *d* = 0.26, and both groups spent significantly more time on all music activities than the less accomplished violinists (*M* = 30.64, 95% CI [22.41, 38.88], range = 13.75–72.88), *p* = .003, *d* = 1.65 and *p* = .014, *d* = 1.17, respectively.

When excluding participants who reported that the diary week was atypical, the pattern of results did not change. The average amount of time spent on all music activities was 50.69 (95% CI [42.94, 58.44], range = 24.00–83.50). The significant difference across groups remained, *F*(2, 18) = 12.99, *p* < .001, η^2^ = .59. Post-hoc follow-up tests revealed that the best violinists (*M* = 57.86, 95% CI [47.58, 68.14], range = 30.00–83.50) and the good violinists (*M* = 58.82, 95% CI [50.95, 66.70], range = 44.00–71.75) spent similar amounts of time engaged in musical activities, *p* = .979, *d* = -0.07, and both groups spent significantly more time on all music activities than the less accomplished violinists (*M* = 26.39, 95% CI [24.15, 28.63], range = 24.00–30.50), *p* = .001, *d* = 2.63, and *p* = .001, *d* = 4.23, respectively.

**Deliberate practice.**

***Original study results.*** Despite defining deliberate practice as teacher-designed activities, Ericsson et al. asked participants to estimate amount of time spent “practicing alone.” There was no code for teacher-designed practice. Ericsson et al.^[S1]^ found that the best violinists (*M* = not reported) and good violinists (*M* = not reported) practiced alone similar amounts during the diary week and that the mean hours of practice alone for the combined best and good violinist group (*M* = 24.3) was significantly higher than that of the less accomplished violinists (*M* = 9.3) at the *p* < .001 level.

***Replication study results.*** Differing from Ericsson et al. (1993), participants in the present study had the additional option of coding their practice sessions as teacher-designed practice. Inspection of the diaries revealed that half the participants used the practice alone code, but did not use the teacher-designed practice code as the primary identifier of the activity at any point during the diary week. The remaining participants used both the practice alone and teacher-designed practice codes to identify their practice activities, either in conjunction (i.e., assigning equal weight to the activity as practice alone and teacher-designed practice) or independently (i.e., assigning some practice sessions or some parts of practice sessions primarily as practice alone and other sessions or parts of sessions as primarily teacher-designed practice).

The likelihood of using the teacher-designed practice code did not differ by group, χ^2^(2) < .001, *p* > .999. Overall, on average, only 3.12 hours were coded as teacher-designed practice (95% CI [1.41, 4.83], range = 0.00–22.75). This amount did not differ by group, χ^2^(2) = 1.00, *p* = .606, η^2^ = .08.

Given that a) the majority of participants primarily used one code, b) there were no differences by group in code use, and c) Ericsson et al.'s code of "practice alone" presumably captured teacher-designed practice as well, we report the sum of activities coded as practice alone, teacher-designed practice, or both practice alone and teacher-designed practice and call these activities "deliberate practice."

We found a significant effect of group for amount of time spent on deliberate practice during the diary week, χ^2^(2) = 17.15, *p* < .001, η^2^ = .41. In line with Ericsson et al.'s ^[S1]^ findings, post-hoc follow up tests revealed that the amount of time spent engaged in deliberate practice during the diary week did not differ significantly between the best violinists (*M* = 21.53, 95% CI [17.88, 25.19], range = 11.75–31.75) and the good violinists (*M* = 22.03, 95% CI [14.48, 29.57], range = 4.25–44.50), *p* = .990, *d* = -0.05. The best violinists and the good violinists reported significantly more time engaged in deliberate practice during the diary week than the less accomplished violinists (*M* = 6.60, 95% CI [4.81, 8.38], range = 2.50–12.00), *p* = .001, *d* = 3.08, and *p* < .001, *d* = 1.59, respectively.

When excluding participants who reported that the diary week was atypical, the pattern of results was unchanged. The main effect of group remained, *F*(2, 18) = 14.41, *p* < .001, η^2^ = .62. Post-hoc follow up tests revealed that the best violinists (*M* = 21.63, 95% CI [17.65, 25.60], range = 13.88–31.75) and the good violinists (*M* = 29.88, 95% CI [21.99, 37.76], range = 15.00–44.50) did not differ significantly, *p* = .104, *d* = -1.06, and that both the best and good violinists practiced significantly more than the less accomplished violinists (*M* = 6.28, 95% CI [3.26, 9.29], range = 2.50–10.00), *p* = .005, *d* = 3.10, and *p* < .001, *d* = 3.03, respectively. Regardless of whether including all participants or only those who reported that the diary week was typical, the pattern of results from the present study replicate the pattern of results from Ericsson et al..

***Daily deliberate practice.***

*Original study results.* Ericsson et al.^[S1]^ report that the best violinists and good violinists practiced similar amounts during the diary week (means and statistical analyses not reported). They report that the mean hours of practice alone for the combined best and good violinist group (3.5 hours per day) was significantly higher than that of the less accomplished violinists (1.3 hours per day). Ericsson et al. analyzed the daily amount of practice alone as a function of the day of the week for the three student groups. Ericsson et al. (1993) state that no main effect or interaction of day of the week was observed (statistical results not reported).

*Replication study results.* We found that the best violinists engaged in deliberate practice with the violin on average for 3.08 hours per day (95% CI [2.55, 3.60], range = 1.68–4.54) during the diary week, the good violinists engaged in deliberate practice with the violin on average for 3.15 hours per day (95% CI [2.07, 4.22], range = 0.61–6.36) during the diary week, and the less accomplished violinists engaged in deliberate practice with the violin on average for 0.94 hours per day (95% CI [0.69, 1.20], range = 0.36–1.71) during the diary week.

When excluding the violinists who reported that the diary week was atypical, we found that the best violinists engaged in deliberate practice with the violin on average for 3.09 hours per day (95% CI [2.52, 3.66], range = 1.98–4.54) during the diary week, the good violinists engaged in deliberate practice with the violin on average for 4.27 hours per day (95% CI [3.14, 5.39], range = 2.14–6.36) during the diary week, and the less accomplished violinists engaged in deliberate practice with the violin on average for 0.90 hours per day (95% CI [0.47, 1.33], range = 0.36–1.43) during the diary week.

We observed an effect of day of the week, *F*(4.52, 158.09) = 3.18, *p* = .012, η_p_^2^ = .08, which was qualified by a significant interaction between day of the week and skill group, *F*(9.03, 158.09) = 2.90, *p* = .003, η_p_^2^ = .14. The interaction was driven by a general trend of the best and good violinists tending to practice more per day earlier in the week than on the weekends while the less accomplished violinists practiced a fairly consistent amount each day throughout the week, see Figure S5 upper panel.

When excluding participants who reported that the diary week was atypical, the pattern of results did not change. We again found a main effect of day of the week, *F*(6, 108) = 2.93, *p* = .011, η_p_^2^ = .14, and a significant group × day of the week interaction, *F*(12, 108) = 2.09, *p* = .023, η_p_^2^ = .19, see Figure S5 lower panel.

*Figure S5.* Amount of daily practice alone plus teacher-designed practice as a function of day of the week by group including all student violinists (upper panel in black), and by group including only participants who reported that the diary week was typical (lower panel in gray). Error bars represent 95% confidence intervals.

***Deliberate practice by time of day.***

*Original study results.* Ericsson et al. ^[S1]^ plotted the percentage of time used for practice alone in 15-minute intervals from 6:00am to 12:00am collapsed across days for each of the three student groups. They observed that the best and good violinists preferred to practice before lunch.

Ericsson et al. next examined the percentage of time each student violinist spent practicing alone for five 2-hour intervals from 10:00am-8:00pm for each day of the week. They found no main effects or interactions with day of the week when comparing the best violinists to the good violinists. However, when combining the best and good violinists into a single group and comparing this group to the less accomplished violinists, they found a main effect of time of day at the *p* < .001 level, and a significant interaction between group (best and good violinists combined vs. teachers) and time of day at the *p* < .05 level. They report that the interaction was due to the best and good violinists practicing the most during 10:00am–2:00pm whereas the less accomplished violinists' practice was distributed evenly throughout the day. They do not report if day of the week and time of day interacted.

*Replication study results.* We observed that the best violinists tended to practice before and after lunch, while the good violinists and the less accomplished violinists tended to practice soon after lunch, see Figure S6.

Similar to Ericsson et al., we found a main effect of time of day, *F*(3.29, 115.10) = 5.82, *p* = .001, η_p_^2^ = .14 and a significant interaction between time of day and group, *F*(6.58, 115.10) = 2.63, *p* = .017, η_p_^2^ = .13 on percentage of time spent on deliberate practice during the diary week. The interaction was due to the best and good violinists practicing the most mid-afternoon whereas the less accomplished violinists' practice was distributed evenly throughout the day, see Figure S7 upper panel. The day of the week did not interact with time of day or with time of day and group, *p*s > .328

*Figure S6.* Proportion of time spent practicing as a function of time of day from 6:00am to midnight for each of the three student groups, including all diaries (black) and only those who reported that the diary week was typical (gray). Error bars represent 95% confidence intervals.

When excluding participants who reported that the diary week was atypical, the pattern of results did not change. We still found a main effect of time of day, *F*(4, 72) = 5.94, *p* < .001, η_p_^2^ = .25 and a significant interaction, *F*(8, 72) = 2.52, *p* = .018, η_p_^2^ = .22. The interaction was due to the best and good violinists practicing the most mid-afternoon whereas the less accomplished violinists' practice was distributed evenly throughout the day, see Figure S7 lower panel. The day of the week did not interact with time of day or with time of day and group, *p*s > .628

*Figure S7.* Proportion of time spent practicing alone as a function of 2-hour intervals for the three student violinist groups for all students (upper panel in black) and for only those who reported that the diary week was typical (lower panel in gray). Error bars represent 95% confidence intervals.

***Duration and frequency of deliberate practice.***

*Original study results.* Ericsson et al. ^[S1]^ observed that the mean duration of practice sessions during the diary week lasted for approximately 80 minutes and did not differ by day or group (statistical results not reported).

Ericsson et al. found that group differences in amount of practice alone reflected differences in the frequency of practice sessions (rather than the duration of practice sessions). Specifically, they found that the number of practice sessions during the diary week did not differ between the best and good violinists (statistical results not reported), but that the average number of practice sessions of the best and good violinists combined (19.5) differed significantly from the number of practice sessions of the less accomplished violinists (7.1) that week at the *p*  < .001 level.

*Replication study results.* The average length of practice sessions (reported as practice alone, teacher-designed practice, or both) in the current experiment was 89.56 minutes (95% CI [77.75, 101.37], range = 34.69–172.50). Similar to Ericsson et al.'s findings, this amount was consistent across days and day and group did not interact, *p*s > .563. However, we observed a significant effect of skill group, *F*(2, 35) = 5.44, *p* = .009, η_p_^2^ = .24. There were no differences between the best violinists (*M* = 91.32, 95% CI [69.32, 113.31], range = 47.00–172.50) and good violinists (*M* = 107.72, 95% CI [84.19, 131.24], range = 34.69–166.88), *p* = .801, *d* = -0.41, or between the best violinists and the less accomplished violinists (*M* = 69.77, 95% CI [61.87, 77.67], range = 45.00–100.00), *p* = .051, *d* = 0.78. The good violinists' practice sessions were significantly longer than the less accomplished violinists' practice sessions, *p* = .010, *d* = 1.22.

Excluding participants who indicated that the diary week was atypical did not change the pattern of results. Average session duration amount remained consistent across days and day and group did not interact, *p*s > .513. The effect of group remained significant, *F*(2, 18) = 7.76, *p* = .004, η_p_^2^ = .46. There were no differences between the best violinists (*M* = 98.51, 95% CI [72.13, 124.90], range = 48.97–172.50) and good violinists (*M* = 128.32, 95% CI [100.19, 156.44], range = 71.79–166.88), *p* = .106, *d* = -0.81, or between the best violinists and the less accomplished violinists (*M* = 69.21, 95% CI [51.62, 86.81], range = 50.00–100.00), *p* = .103, *d* = 0.91. The good violinists' practice sessions were significantly longer than the less accomplished violinists' practice sessions, *p* = .003, *d* = 2.02.

In addition to the group differences we found for duration, we also found that the number of sessions differed by group, χ^2^(2) = 15.52, *p* < .001, η^2^ = .38. Similar to Ericsson et al., we found that the best violinists (*M* = 16.25, 95% CI [11.97, 20.53, range = 6–30) held a similar number of practice sessions as the good violinists (*M* = 12.31, 95% CI [8.75, 15.87], range = 2–23), *p* = .229, *d* = 0.58. Both the best and good violinists engaged in more practice sessions during the diary week than the less accomplished violinists (*M* = 5.54, 95% CI [4.27, 6.81], range = 3–10), *p* < .001, *d* = 2.03, and *p* = .016, *d* = 1.43, respectively.

When excluding participants who indicated that the diary week was atypical, the pattern of results was unchanged. We continued to observe a main effect of group, *F*(2, 18) = 5.29, *p* = .016, η^2^ =. 37. The best violinists (*M* = 15.22, 95% CI [10.50, 19.94], range = 6–30) held a similar number of practice sessions as the good violinists (*M* = 14.71, 95% CI [10.55, 18.88], range = 6–23), *p* = .984, *d* = 0.08. Both the best and good violinists engaged in more practice sessions during the diary week than the less accomplished violinists (*M* = 5.20, 95% CI [3.40, 7.00], range = 3–7), *p* = .018, *d* = 1.80, and *p* = .033, *d* = 2.30, respectively.

**Relevant and frequent activities.**

***Original study results.*** Ericsson et al. ^[S1]^ report that of the activities rated highly relevant to improving on the violin, only two—practice alone and sleep—exceeded 5 hours per week on average during the diary week.

***Replication study results.*** We found that practice alone with the violin, sleep, practice with others, and music theory, were all rated highly relevant and exceeded 5 hours per week. See Table S5.

| Table S5  *Time Spent on Activities During the Diary Week That Were Rated Highly Relevant and Exceeded 5 Hours Per Week on Average* | | | | | | | | |
| --- | --- | --- | --- | --- | --- | --- | --- | --- |
|  |  | All Diaries (*n* = 38) | | | | | | |
| Activity |  | Best  Violinists |  | Good  Violinists |  | Less Accomplished Violinists |  | All Student Violinists |
|  |  | *M* [lower, upper]  Range |  | *M* [lower, upper]  Range |  | *M* [lower, upper]  Range |  | *M* [lower, upper]  Range |
| Practice  Alone |  | 16.34 [13.31, 19.36]  7.00–25.50 |  | 19.12 [11.27, 26.97]  2.75–44.50 |  | 5.17 [3.05, 7.29]  0.00–12.00 |  | 13.47 [10.00, 16.94]  0.00–44.50 |
| Deliberate  Practice |  | 21.53 [17.88, 25.19]  11.75–31.75 |  | 22.03 [14.48, 29.57]  4.25–44.50 |  | 6.60 [4.81, 8.38]  2.50–12.00 |  | 16.59 [12.94, 20.24]  2.50–44.50 |
| Practice  w/ Others |  | 8.19 [4.31, 12.07]  0.00–22.00 |  | 6.79 [4.07, 9.50]  0.75–16.25 |  | 3.80 [3.10, 4.50]  1.50–6.50 |  | 6.21 [4.58, 7.83]  0.00–22.00 |
| Music  Theory |  | 6.40 [3.13, 9.66]  0.00–18.63 |  | 6.58 [2.03, 11.13]  0.00–24.50 |  | 7.40 [3.35, 11.45]  0.00–29.50 |  | 6.80 [4.54, 9.07]  0.00–29.50 |
| Sleep |  | 55.11 [52.29, 57.94] 46.00–62.50 |  | 57.71 [53.85, 61.58] 41.17–72.50 |  | 56.80 [52.17, 61.42]  46.75–77.75 |  | 56.58 [54.37, 58.79]  41.17–77.75 |
|  |  | Diaries Where the Violinists Reported the Week was Typical (*n* = 21) | | | | | | |
|  |  | Best  Violinists |  | Good  Violinists |  | Less Accomplished Violinists |  | All Student Violinists |
|  |  | *M* [lower, upper]  Range |  | *M* [lower, upper]  Range |  | *M* [lower, upper]  Range |  | *M* [lower, upper]  Range |
| Practice  Alone |  | 17.23 [14.26, 20.19]  12.00–25.50 |  | 25.16 [14.70, 35.62]  3.50–44.50 |  | 4.56 [1.08, 8.05]  0.00–10.00 |  | 16.86 [11.92, 21.79]  0.00–44.50 |
| Deliberate  Practice |  | 21.63 [17.65, 25.60] 13.88–31.75 |  | 29.88 [21.99, 37.76] 15.00–44.50 |  | 6.28 [3.26, 9.29]  2.50–10.00 |  | 20.72 [15.79, 25.65]  2.50–44.50 |
| Practice  w/ Others |  | 9.61 [5.17, 14.05]  2.00–22.00 |  | 6.82 [3.13, 10.51]  1.25–16.25 |  | 3.70 [3.02, 4.38]  5.00–5.00 |  | 7.27 [4.86, 9.68] 1.25–22.00 |
| Music  Theory |  | 5.33 [1.81, 8.85]  0.00–18.63 |  | 6.39 [0.24, 12.55]  0.00–24.25 |  | 5.60 [3.30, 7.90]  2.50–9.50 |  | 5.75 [3.26, 8.24]  0.00–24.25 |
| Sleep |  | 54.90 [51.26, 58.54] 46.00–62.50 |  | 57.27 [51.61, 62.93] 41.17–65.50 |  | 59.25 [49.94, 68.56] 52.00–77.75 |  | 56.73 [53.54, 59.92] 41.17–77.75 |
| Note. lower = 95% confidence interval lower bound. upper = 95% confidence interval upper bound. Deliberate Practice = practice alone with the violin + teacher-designed practice with the violin. Practice w/ Others = Practice with others. | | | | | | | | |

Practice with others did not differ by group neither when examining all diaries, χ^2^(2) = 2.68, *p* = .262, η^2^ = .13, nor when excluding diaries recorded as atypical, χ^2^(2) = 2.92, *p* = .232, η^2^ = .18. Similarly, music theory did not differ by group neither when examining all diaries, *F*(2, 35) = 0.07, *p* = .934, η^2^ < .01, nor when excluding diaries recorded as atypical, *F*(2, 18) = 0.06, *p* = .941, η^2^ < .01.

**Sleep.**

***Original study results.*** Ericsson et al. suggest that sleep is necessary for recovering from practice activities. They measure sleep in two ways (weekly estimate and diary log) and conduct a large number (14) of tests on these two measures.

*Sleep between the best and good violinists during the diary week.* Ericsson et al.^[S1]^ found no difference in amount of sleep during the diary week between the best and good violinists (statistical results not reported).

*Sleep between the best and good violinist combined and the less accomplished violinists during the diary week*. Ericsson et al.^[S1]^ observed that the average amount of sleep for the best and good violinists combined (60.0 hours) during the diary week was significantly longer than for the less accomplished violinists (54.6) at the *p* < .05 level. They suggested that the difference in sleep is related to the difference in practice amounts.

*Sleep between the best and good violinists by day of the week during the diary week.* Ericsson et al. ^[S1]^ conducted an ANOVA on the amount of sleep per day of the week and found no main effect of day or interaction between day of the week and skill group (best and good violinists).

*Sleep between the best and good violinists combined and the less accomplished violinists by day of the week during the diary week.* Ericsson et al. ^[S1]^ conducted an ANOVA on the amount of sleep per day of the week and found no main effect of day or interaction between day of the week and skill group (best and good violinists combined as one group and the less accomplished violinists).

Ericsson et al. next examined napping defined as sleep episodes between 9:00am and 9:00pm separated from nighttime sleep by at least one hour. They found that the best and good violinists did not differ from each other, but that the average hours of napping during the week of the best and good violinists combined (*M* = 2.8) was significantly greater than the nap time of the less accomplished violinists (*M* = .09) at the *p* < .05 level.

Ericsson et al. then examined napping as a function of day of the week by group. They found a significant interaction between day of the week and group at the p < .05 level when comparing the best and good violinists (combined as a single group) with the less accomplished violinists.

***Replication study results.*** We found no significant difference in amount of sleep during the diary week across groups, *F*(2, 35) = 0.43, *p* = .653, η^2^ = .02. All groups slept approximately 56.58 hours per week 95% CI [54.37, 58.79], range = 41.17–77.75. This result did not change when only examining students indicating that the diary week was typical, *F*(2, 18) = 0.55, *p* = .588, η^2^ = .06. See Figure S8.


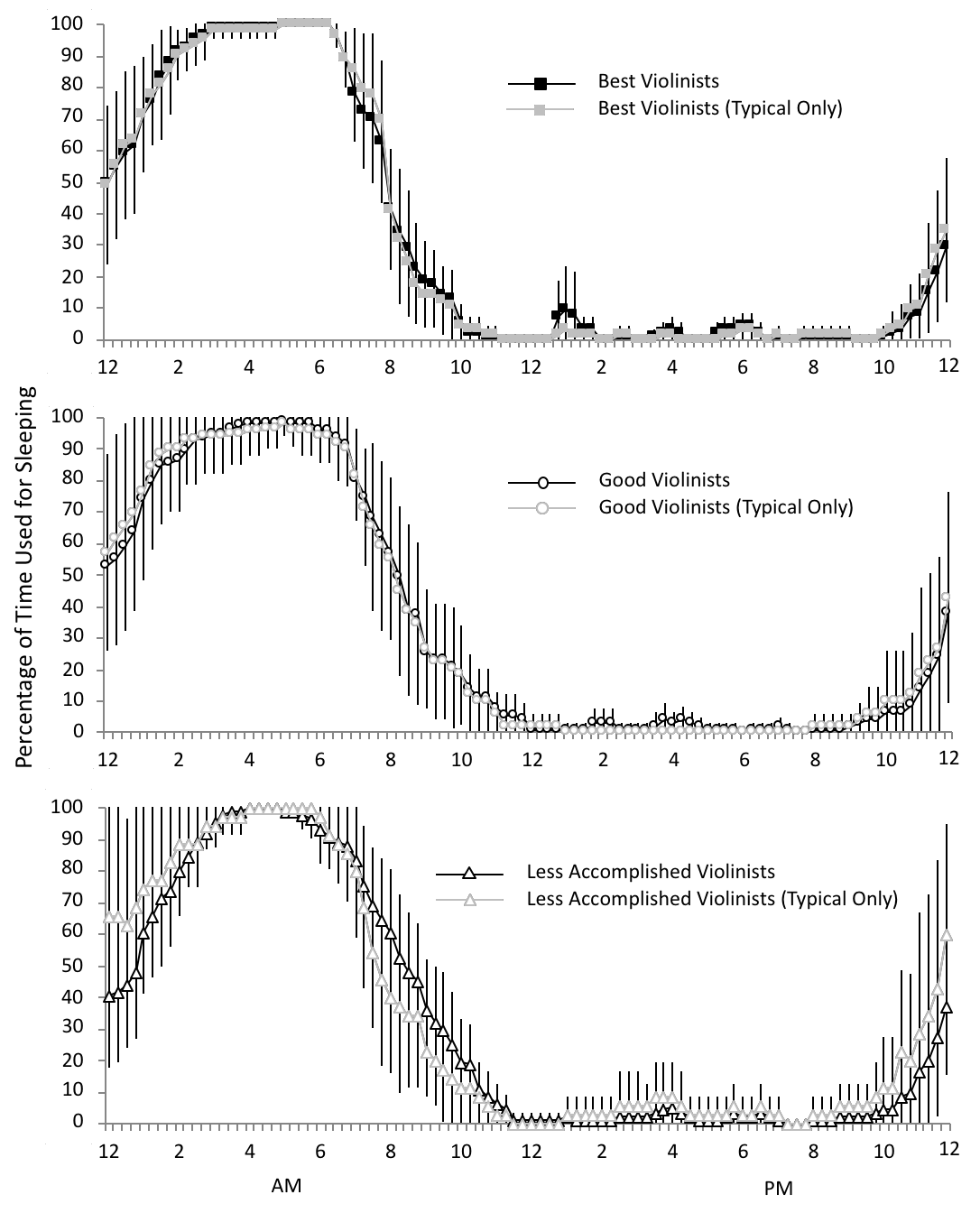
Figure S8. Percentage of time spent sleeping for each student group as a function of time of day. Gray indicates results for only those participants who reported that the diary week was typical. Error bars represent 95% confidence intervals.

In the present study, we found a main effect of day of the week, *F*(6, 210) = 3.81, *p*  = .001, η_p_^2^ = .10, but no interaction with skill group, *p* = .341. Participants slept the least on Wednesdays and the most on Saturdays. See Figure S9 upper panel. The results did not change when only including participants who reported that the diary week was typical: main effect of day, *F*(6, 108) = 3.26, *p* = .005, η_p_^2^ = .15, no interaction with skill group, *p* = .572. See Figure S9 lower panel.

*
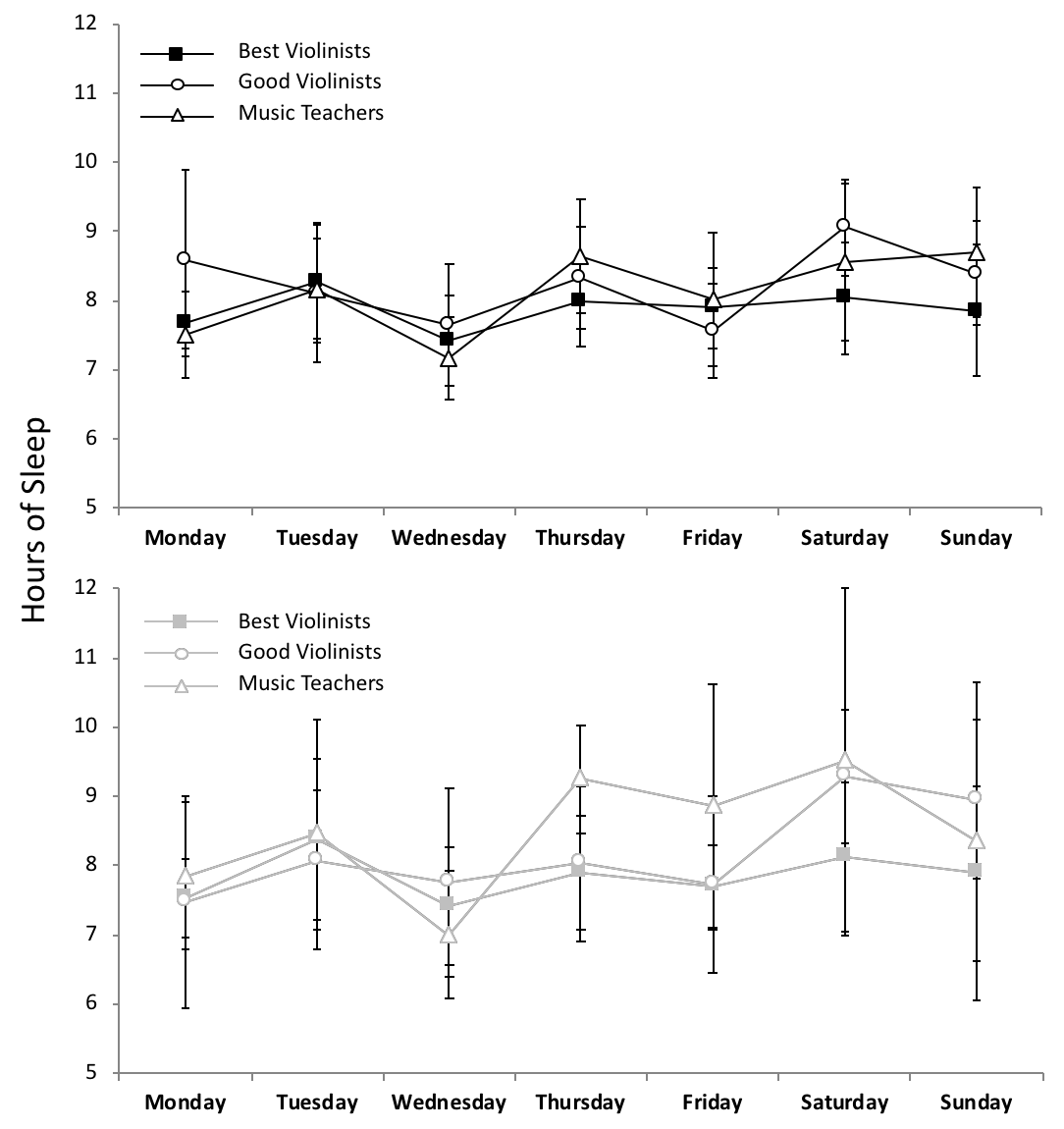
Figure S9.* Hours of sleep by group by day of the week for all student violinists (upper panel in black) and for only those participants who reported that the diary week was typical (lower panel in gray). Error bars represent 95% confidence intervals.

We did not observe any differences among the groups in napping, χ^2^(2) = 0.80, *p* = .672, η^2^ < .01. Most participants did not nap at all. The average amount of napping was 1.14 hours across all student violinists (95% CI [0.60, 1.69], range = 0.00–7.25). This pattern did not change when only examining participants who reported that the diary week was typical, χ^2^(2) = 5.73, *p* = .057, η^2^ = .28, *M* = 0.83, 95% CI [0.10, 1.57], range = 0.00–7.25.

We also observed a significant interaction between day of the week and group, *F*(7.35, 128.56) = 2.17, *p* = .038, η_p_^2^ = .11. However, both Mauchley's test was significant and most days contained non-normal distributions. When applying a stricter lower-bound correction due to the non-normal distributions, the interaction is not significant, *F*(2, 35) = 2.17, *p* = .129. See Figure S10 upper panel. This pattern holds when examining only those entries where the participants reported the diary week was typical (where normal distributions are observed),: *F*(4.69, 42.23) = 2.96, *p* = .024, η_p_^2^ = .25; *F*(2, 18) = 2.96, *p* = .077. See Figure S10 lower panel.

*
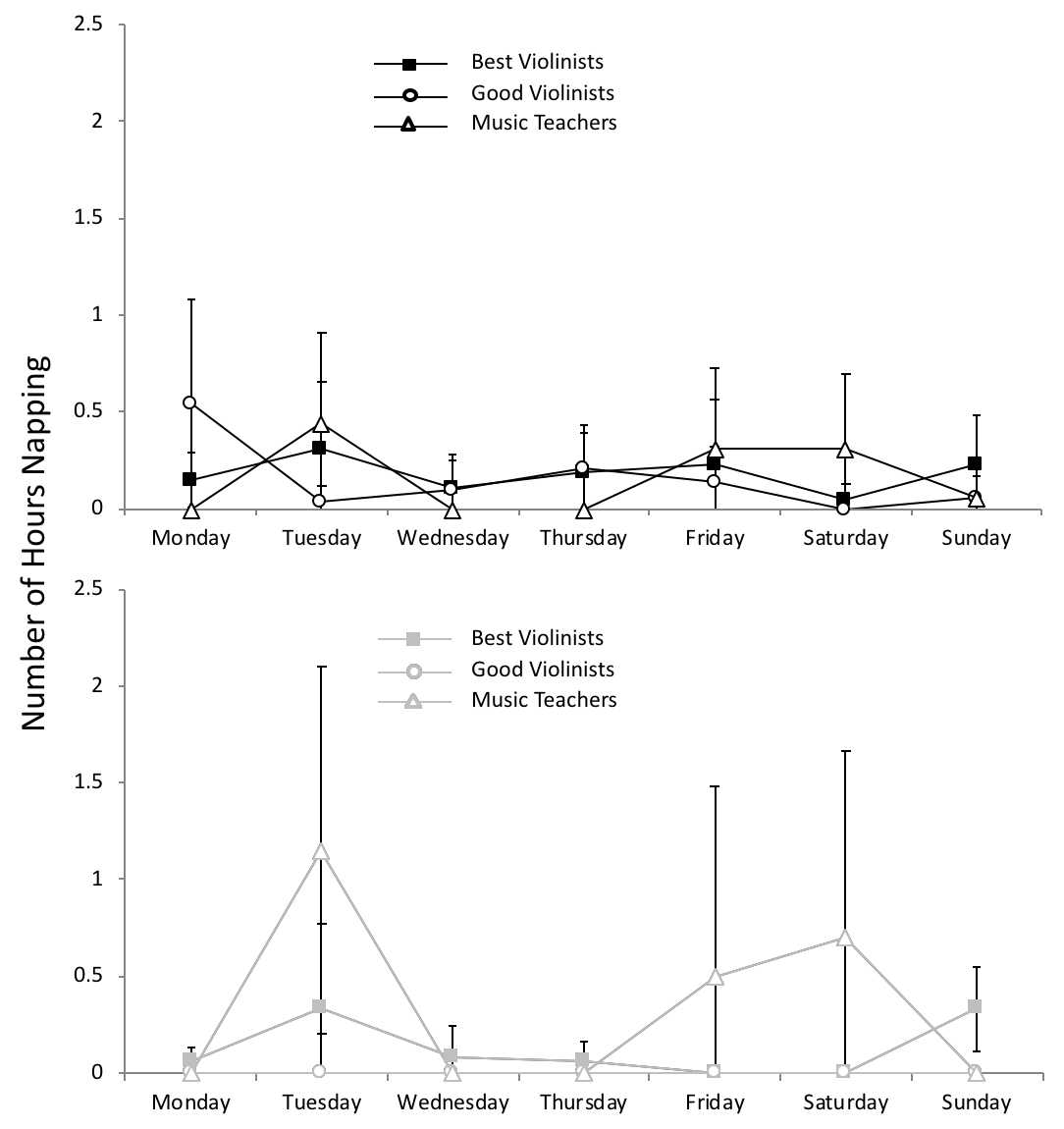
Figure S10.* Hours spent napping by day of the week and group for all student violinists (upper panel in black) and for participants who reported the diary week was typical (lower panel in gray). Error bars represent 95% confidence intervals.

**Leisure.**

***Original study results.*** Ericsson et al. suggest that in addition to sleep, leisure time also aids in recovery from practice. They performed an ANOVA on daily amount of leisure by day and by group. Contrary to the leisure-as-practice-recovery hypothesis, they found that the best violinists spent significantly less time on leisure activities per day (*M* = 3.5) than the good violinists (*M* = 4.7) at the *p* < .05 level, and no significant difference between the two high-practicing groups combined (best and good violinists, *M* = 4.1) and the less accomplished violinists (*M* = 4.0) who practice less. Furthermore, they also report a *negative* association between leisure time and music-related activities at the p *<* .05 level for all the student violinists. Despite these findings not supporting the leisure-as-practice-recovery hypothesis, they interpret the results as "consistent with the hypothesis that activities other than sleep can provide necessary rest" (p. 377).

***Replication study results.*** We found a main effect of group, *F*(2, 35) = 4.10, *p* = .025, η_p_^2^ = .19. The differences were driven by the good violinists (*M* = 2.27, 95% CI [1.51, 3.03], range = 0.04–4.96) engaging in numerically more hours of daily leisure than the best violinists (*M* = 1.25, 95% CI [0.70, 1.81], range = 0.04–3.45), *p* = .067, *d* = -0.07, and significantly more than the less accomplished violinists (*M* = 1.15, 95% CI [0.70, 1.60], range = 0.16–3.18), *p* = .035, *d* = 1.02. The best violinists and less accomplished violinists engaged in leisure time similarly, *p* = .968, *d* = 0.12. As with Ericsson et al., we observed a main effect of day of the week, *F*(3.97, 138.86) = 5.12, *p* = .001, η_p_^2^ = .13, with more leisure time spent on the weekends, but no significant interaction with group, *p* = .465. See Figure S11 upper panel. The correlation between leisure time and time spent on musical activities was negative but not significant, *r*(36) = -.25, *p* = .134.

When excluding participants who reported that the diary week was atypical, the pattern of results was similar. We still observed a main effect of group, *F*(2, 18) = 5.45, *p* = .014, η_p_^2^ = .38. The good violinists (*M* = 2.35, 95% CI [1.22, 3.49], range = 0.36–4.96) engaged in significantly more hours of daily leisure than the best violinists (*M* = 0.89, 95% CI [0.47, 1.31], range = 0.04–1.93), *p* = .024, *d*  = -1.40, and significantly more than the less accomplished violinists (*M* = 0.73, 95% CI [0.43, 1.03], range = 0.21–1.00), *p* = .032, *d* = 1.47. The best violinists and less accomplished violinists engaged in leisure time similarly, *p* = .954, *d*  = 0.31. We did not observe a main effect of day of the week, *F*(2.98, 53.60) = 2.72, *p* = .054, η_p_^2^ = .13, though numerically more leisure time spent on the weekends. However, we observed a marginally significant interaction, *F*(5.96, 53.60) = 2.33, *p* = .046, η_p_^2^ = .21. The interaction was driven by the good violinists engaging in substantially more leisure time than the other two groups on the weekend. See Figure S11 lower panel.

*
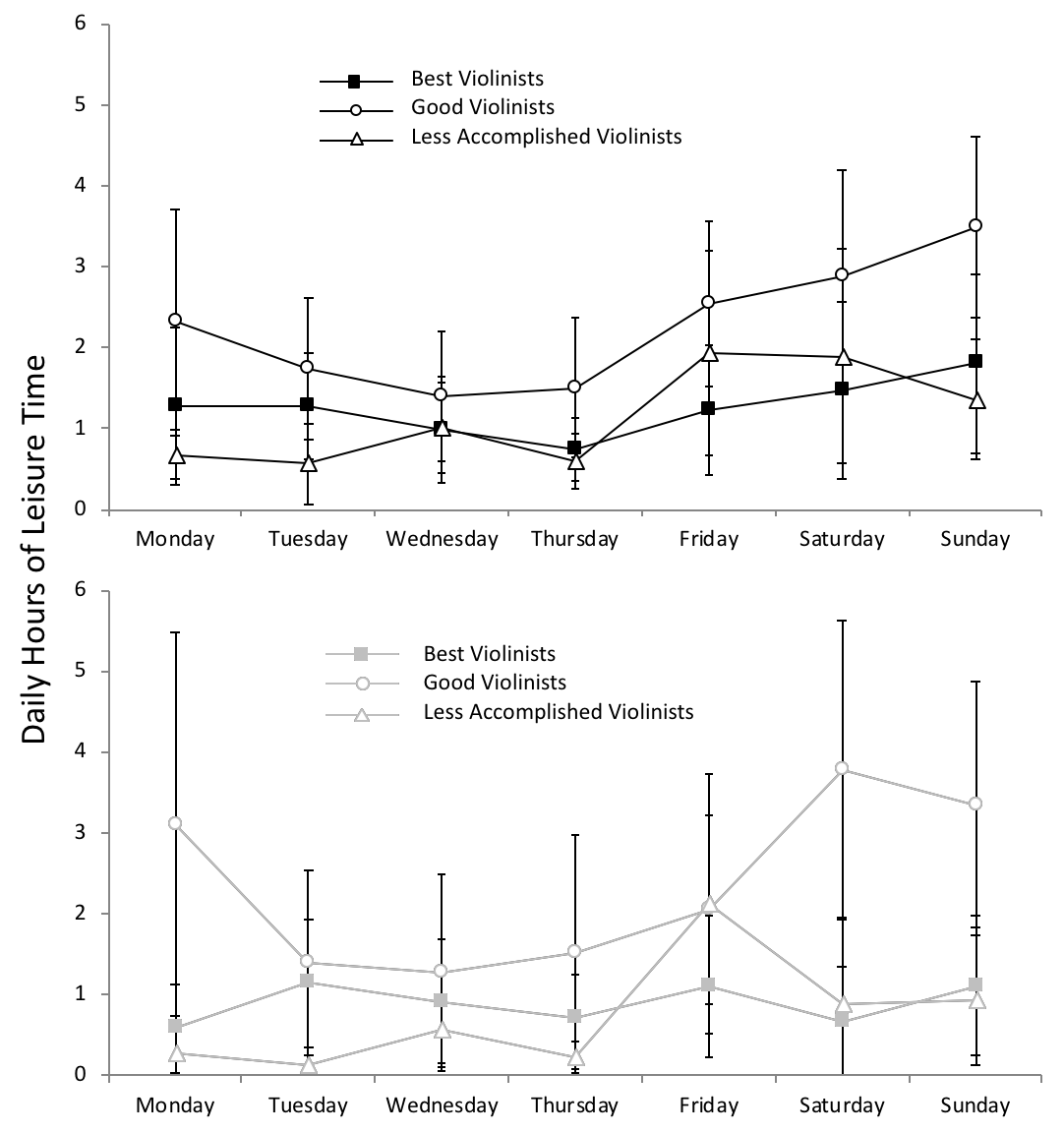
Figure S11.* Hours spent in leisure by day of the week and group for all student violinists (upper panel in black) and for only those participants who reported that the diary week was typical (lower panel in gray). Error bars represent 95% confidence intervals.

We added social media/email as a category of activities. Social media might function as leisure time for students. We re-ran these analyses adding social media/email with other leisure activities. Including social media activities as leisure time, we no longer observe a main effect of group, *F*(2, 35) = 1.74, *p* = .191, η_p_^2^ = .09. Students spent about 1.5 to 2.5 hours per day on leisure and social media/emailing. The main effect of day remains, *F*(6, 210) = 2.96, *p* = .009, η_p_^2^ = .08, with the most leisure on weekends. Day of the week and group do not significantly interact, *p* = .599. See Figure S12 upper panel. When excluding violinists who reported that the diary week was atypical, no main effects or interactions were observed, all *p*s > .083. See Figure S12 lower panel.

*
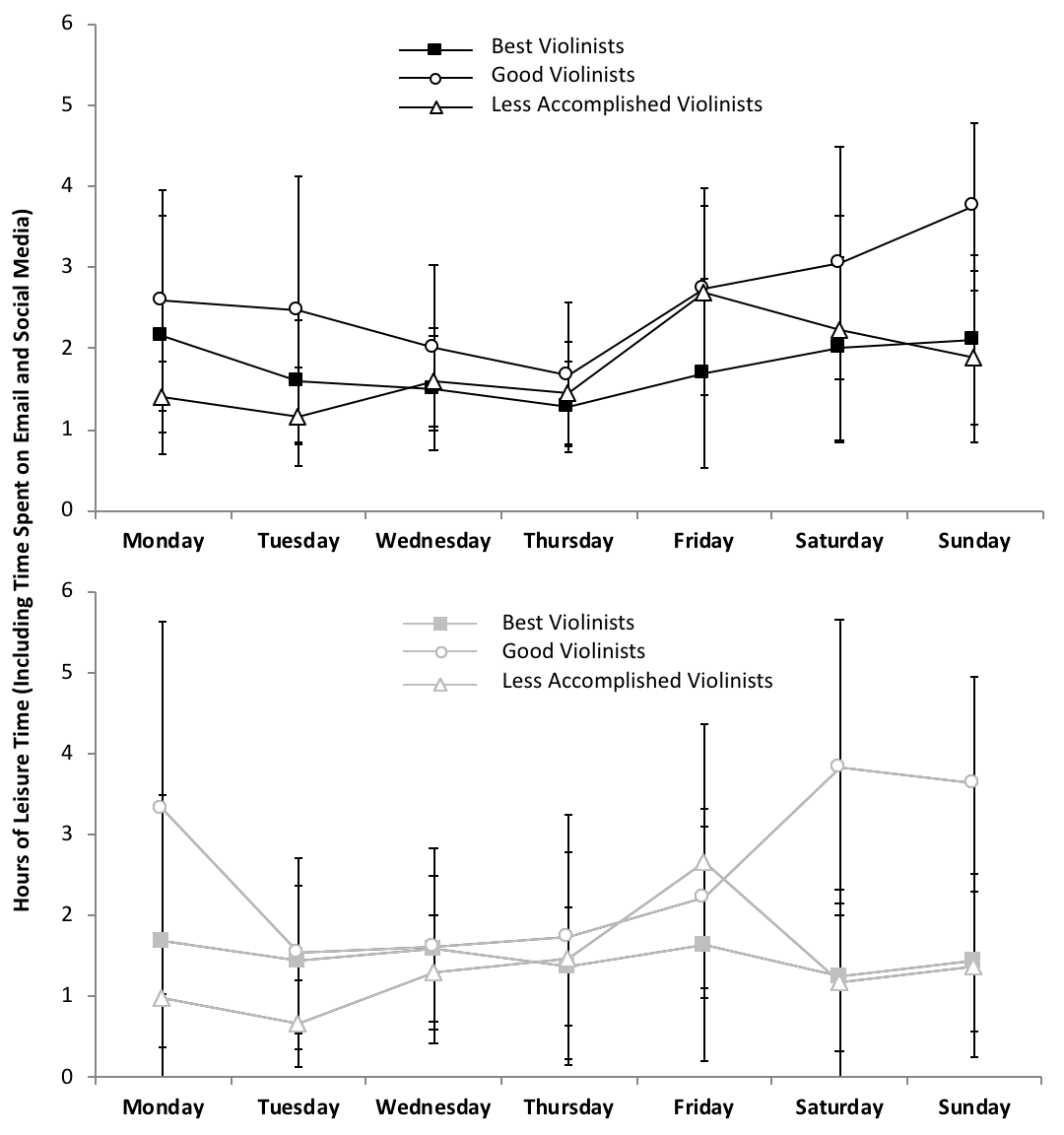
Figure S12.* Hours spent in leisure time, emailing, and using social media as a function of day of the week and group for all student violinists (upper panel in black) and for only those participants who reported that the diary week was typical (lower panel in gray). Error bars represent 95% confidence intervals.

**Estimates of Current Time Spent in Musical and Everyday Activities**

**Original study results.** Ericsson et al. analyzed student violinists’ estimates of current time spent on practice alone, sleep, and leisure. They do not report any descriptive statistics for the best violinists or the good violinists. They report the mean for the best and good violinists combined for practice alone (*M* = 29.8), but not for sleep or leisure. They report the less accomplished violinists’ mean estimate of practice alone (*M* = 13.4), but not for sleep or leisure.

**Replication study results.** Table S6 shows the descriptive statistics for the estimates of current weekly time spent on activities analyzed by Ericsson et al. (1993)—practice alone, sleep, and leisure—as well as teacher-designed practice and the combination of leisure and social media/email added in the present study.

| Table S6  *Estimates of Current Time Spent Activities During a Typical Week* | | | | | | |
| --- | --- | --- | --- | --- | --- | --- |
| Activity |  | Best Violinists |  | Good Violinists |  | Less Accomplished |
|  |  | *M* [lower, upper]  range |  | *M* [lower, upper]  range |  | *M* [lower, upper]  range |
| Practice Alone |  | 22.69 [17.65, 27.74]  8.00–40.00 |  | 30.65 [27.04, 34.27]  20.00–45.50 |  | 9.58 [6.55, 12.61] 2.00–18.00 |
| Teacher-designed Practice |  | 10.23 [4.82, 15.64]  0.00–28.00 |  | 14.88 [7.77, 22.00]  0.00–35.00 |  | 4.46 [1.96, 6.96] 0.00–15.00 |
| Sleep |  | 46.08 [41.22, 50.94] 21.00–56.00 |  | 44.15 [41.37, 46.94] 38.00–56.00 |  | 45.15 [42.42, 47.89]  35.00–51.00 |
| Leisure |  | 7.92 [5.06, 10.78]  2.00–21.00 |  | 8.54 [4.36, 12.71]  1.00–25.00 |  | 7.00 [5.13, 8.87]  2.00–12.00 |
| Leisure +  Social Media/Email |  | 17.85 [12.13, 23.57]  4.50–35.00 |  | 15.88 [9.77, 22.00]  2.50–35.00 |  | 17.08 [12.49, 21.67]  5.00–31.00 |

Note. lower = 95% confidence interval lower bound. upper = 95% confidence interval upper bound.

**Practice alone and Teacher-designed practice.**

***Original study results.*** For practice alone, Ericsson et al. report that the best violinists and the good violinists did not differ significantly (statistics for difference not reported). They report that the best and good violinists combined as a single group practiced alone more than the less accomplished violinists at the *p* < .001 level. Ericsson et al. did not ask participants to estimate teacher-designed practice.

Ericsson et al. compared the estimates of current weekly practice alone with the diary logs of practice alone. They found that the two measures of practice alone were highly correlated (*r* = .74, *p* < .001).

Ericsson reported that participants estimated engaging in significantly more practice alone per week than they logged at the *p* < .001 level, but importantly, this overestimate did not differ across groups.

***Replication study results.*** Participants generally estimated more time spent practicing alone than engaging in teacher-designed practice, *F*(1, 36) = 37.99, *p* < .001, η_p_^2^ = .51. This difference in estimates was consistent across groups, χ^2^(2) = 5.15, *p* = .076, η^2^ = .15. Planned comparisons further indicated that the difference was similar for the best violinists (*M* = 12.46, 95% CI [7.38, 17.54], range = 2.00–38.00) and the good violinists (*M* = 15.77, 95% CI [6.80, 24.74], range = -7.00–38.50), *t*(18.98) = -0.63, *p* = .537, *d* = -0.26.

The two measures of practice alone (diary and estimates) in the current study were moderately correlated, *r*(36) = .53, *p* = .001. This lower correlation might be due to almost half the participants using the teacher-designed practice code instead of or in combination with the practice alone code sometimes during the week, even when presumably practicing alone. Next, we examined the correlations between estimates of current weekly teacher-designed practice and diary-logged practice. In line with our observation that the majority of participants coded practice as practice alone over teacher-designed practice, the correlations between estimates of teacher-designed practice and logged teacher-designed practice, *r*(36) = .32, *p* = .050, and between estimates of teacher-designed practice and practice coded as practice alone or teacher-designed practice, *r*(36) = .03, *p* = .839, were low and non-significant. When examining the correlation between estimates of practice alone and logged practice time coded as practice alone and/or teacher-designed practice, the correlation was *r*(36) = .65, *p* < .001.

Similar to Ericsson et al., we found that the estimates of current typical practice alone were significantly higher than amount of practice alone logged during the diary week, *F*(1, 35) = 19.62, *p* < .001, η_p_^2^ = .36, and that this overestimate did not differ across groups, *F*(2, 35) = 1.45, *p* = .249, η_p_^2^ = .08. The consistent overestimation of practice alone across groups remained when comparing estimates of practice alone to practice alone during the diary week for those who reported that the diary week was typical [main effect of measure: *F*(1, 18) = 5.04, *p* = .038, η_p_^2^ = .22, no interaction with group: *F*(2, 18) = 0.07, *p* = .936, η_p_^2^ = .01].

We also compared estimates of teacher-designed practice to combined practice alone and teacher-designed practice during the diary week. We used the combination of practice activities as a comparison because many participants appeared to code teacher-designed practice as practice alone. Again, we found an overestimation, *F*(1, 35) = 7.12, *p* = .011, η_p_^2^ = .17, that did not differ by group, *F*(2, 35) = 0.98, *p* = .384, η_p_^2^ = .05. The same pattern was observed when only examining those who reported that the diary week was typical [main effect of measure: *F*(1, 18) = 9.89, *p* = .006, η_p_^2^ = .35, no interaction with group: *F*(2, 18) = 1.54, *p* = .241, η_p_^2^ = .15].

**Sleep.**

***Original study results.*** Ericsson et al. report that for sleep, the best violinists and the good violinists did not differ significantly (statistics not reported). They report that the best and good violinists combined estimated significantly more sleep than the less accomplished violinists, at the *p* < .05 level.

Ericsson et al. compared the estimates of current typical sleep to the diary-logged amounts of sleep and found that the estimates were significantly lower than the diary-logged amounts at the *p* < .01 level, but that there was no difference in underestimation across the groups (i.e., no interaction). Ericsson et al. found a moderate correlation between estimated sleep and diary-logged sleep (*r* = .49, *p* < .01).

***Replication study results.*** We did not observe a main effect of sleep amount estimates, *F*(2, 36) = 0.27, *p* = .762, η^2^ = .02. See Table S6.

Similar to Ericsson et al., we found that the estimates of sleep were significantly lower than the diary-logged amounts, *F*(1, 35) = 64.62, *p* < .001, η_p_^2^ = .65, and that there was no interaction with group, *F*(2, 35) = 0.86, *p* = .432, η_p_^2^ = .05. This pattern held when excluding participants who reported that the diary week was atypical [main effect of measure: *F*(1, 18) = 49.59, *p* < .001, η_p_^2^ = .73, no interaction with group: *F*(2, 18) = 3.11, *p* = .069, η_p_^2^ = .26]. We did not find that the estimates for sleep and the diary-logged amounts of sleep were correlated, *r*(36) = .18, *p* = .293.

**Leisure.**

***Original study results.*** Ericsson et al. report that for leisure, the best violinists and the good violinists did not differ significantly (statistics not reported). They report that the best and good violinists combined estimated significantly lower amounts of leisure than the less accomplished violinists at the *p* < .05 level.

Ericsson et al. compared the estimates of leisure time with the diary logs and found that estimates were significantly lower than diary-logged amounts at the *p* < .001 level. They also found a significant interaction between the measure differences and the difference between the best and good violinists. Ericsson et al. reported that there was not a significant correlation between estimates of leisure time and diary-logged leisure time.

***Replication study results.*** We found no effect of group on estimates of typical amounts of leisure, χ^2^ = 0.06, *p* = .971, η^2^ = .01. There was also no effect when combining estimates of leisure activities with estimates of social media/email, *F*(2, 36) = 0.12, *p* = .884, η^2^ < .01.

Similar to Ericsson et al., we found that the estimates were significantly lower than the diary-logged amounts, *F*(1, 35) = 11.10, *p* = .002, η_p_^2^ = .24, and that this difference interacted with group, *F*(2, 35) = 3.70, *p* = .035, η_p_^2^ = .17. This interaction was driven by the good violinists estimating similar amounts of leisure time as the best violinists and less accomplished violinists but logging almost twice as many hours as their estimates, while the other two groups logged approximately one hour more than their estimates. This pattern of results was similar when excluding participants who reported that the diary week was atypical [main effect of measure: *F*(1, 18) = 8.57, *p* = .009, η_p_^2^ = .32; interaction: *F*(2, 18) = 8.82, *p* = .002, η_p_^2^ = .50. We observed a significant correlation, *r*(36) = .57, *p* < .001.

When combining leisure time with social media time/email, there was no difference between estimates and logged time, *F*(1, 35) = 1.37, *p* = .249, η_p_^2^ = .04, and no interaction with group, *F*(2, 35) = 1.62, *p* = .212, η_p_^2^ = .09. However, an interaction emerged when only examining students who indicated that the diary week was typical, *F*(2, 18) = 5.56, *p* = .013, η_p_^2^ = .38. This was driven by the best violinists and less accomplished violinists overestimating this time.

**Additional Measures Administered Following the** **Replication**

**Awareness and beliefs.** Overall, over half (58%) of the violin students were familiar with "deliberate practice." Eighty-four percent were familiar with Malcolm Gladwell's "10,000-hour rule" that was inspired by the deliberate practice theory. Only three student violinists (all from the less accomplished group) were unaware of either deliberate practice or the "10,000-hour rule." Despite the overwhelming familiarity, only 32% of the student violinists believed that with 10,000 hours of practice anyone could become an expert at anything.

On average, the student violinists attributed 42% of their own performance level on the violin to "nature" (i.e., innate abilities/traits) and 58% to "nurture" (i.e., experience/environmental factors). These proportions did not differ by group (best violinists: *M*_nurture_ = 52.50%, 95% CI_nurture_ [43.20%, 61.80%], range_nurture_ = 35%–80%; good violinists: *M*_nurture_ = 61.15%, 95% CI_nurture_ [51.79%, 70.51%], range_nurture_ = 35–90%; less accomplished violinists: *M*_nurture_ = 60.92%, 95% CI_nurture_ [47.37%, 74.48%], range_nurture_ = 20–99% ), *F*(2, 35) = 0.75, *p* = .480, η^2^ = .04. Unsurprisingly, the extent to which students endorsed having a growth mindset of talent—believing that one's talent can improve with effort—was significantly correlated with the proportion of their violin performance level they attributed to "nurture" rather than innate factors, *r*(36) = .61, *p* < .001.

Overall, the correlation between "nurture" attributions and the amount the students overestimated their current amounts of practice alone was non-significant, *r*(36) = .01, *p* = .969. However, this relationship was qualified by a significant interaction with group, Δ*R*^2^ = .17, *p* = .037. For the best violinists, the more they indicated believing their performance on the violin was attributable to nurture, the more they overestimated their current practice alone. For the good violinists, the more they attributed their performance on the violin to nurture, the less they overestimated their practice alone. The less accomplished violinists had very little estimation bias and there was no relationship with nurture attributions. See Figure S13.

*
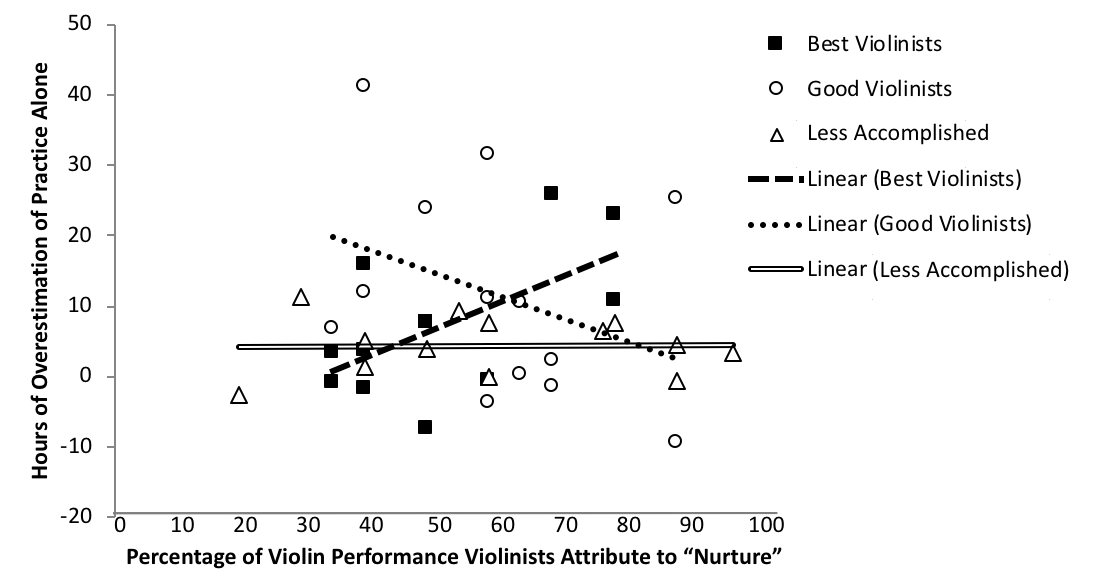
Figure S13.* Overestimation of practice as a function of group and the extent to which one attributes one's violin performance to "nurture" rather than "nature."

The relationship between student violinists' growth mindset of talent and the amount they overestimated their current amounts of practice alone was not significant, *r*(36) = .20, *p* = .239. The lack of relationship between mindsets and overestimations did not differ by skill group, Δ*R*^2^ < .01, *p* = .857.

**Cognitive abilities.** Comparisons of the best and good violinists were conducted on measures of cognitive abilities. The less accomplished were not included because they were comprised of students at a university rated as most competitive. Therefore, despite having lower music performance, they were likely to have greater cognitive abilities by virtue of the high SAT/ACT entrance requirements. We thus only compared the music conservatory students (best and good violinists) on these measures. As can be seen in Table S7, the best violinists' scores were numerically, but not significantly, higher than the good violinists for all abilities. Discriminant function analysis including all four measures successfully predicted 80% of group membership (10 of the 12 best violinists correctly predicted as best violinists; 10 of the 13 good violinists correctly predicted as good violinists). However, neither this model nor any other combination of abilities yielded a significant model, all *p*s > .146.

| Table S7  *Comparison of Cognitive Abilities Between Best and Good Violinists* | | | | | | | | | | | | | | | | | | |
| --- | --- | --- | --- | --- | --- | --- | --- | --- | --- | --- | --- | --- | --- | --- | --- | --- | --- | --- |
|  |  | Best Violinists | | | | |  | Good Violinists | | | | |  | Comparison | | | | |
| Ability |  | *M* |  | 95% CI |  | Range |  | *M* |  | 95% CI |  | Range |  | *t* |  | *p* |  | *d* |
| Working memory capacity |  | .84 |  | [.75, .92] |  | .54–1.00 |  | .69 |  | [.58, .80] |  | .35–.99 |  | 1.98 |  | .060 |  | 0.88 |
| Fluid intelligence |  | .72 |  | [.66, .78] |  | .56–.88 |  | .66 |  | [.59, .74] |  | .35–.94 |  | 1.14 |  | .266 |  | 0.51 |
| Cognitive processing speed |  | .56 |  | [.50, .62] |  | .37–.76 |  | .52 |  | [.46, .59] |  | .32–.68 |  | 0.93 |  | .362 |  | 0.38 |
| Basic music ability |  | .88 |  | [.84, .93] |  | .75–1.00 |  | .86 |  | [.83, .89] |  | .77–.95 |  | 0.78 |  | .432 |  | 0.30 |
| Note. 95% CI = 95% confidence interval. Numbers in brackets represent the lower and upper bounds of the confidence interval. | | | | | | | | | | | | | | | | | | |

**Changes from Initial Preregistration**

- Some planned ANOVAs/*t*-tests violated the homogeneity of variance assumption. Kruskal-Wallis tests and Welch's *t*-tests were used instead.
- We were previously unclear on a particular statistical technique used by Ericsson et al. (1993) (described in Hypothesis 6 Statistical Technique, first bullet point). We later became clear on the technique used and so follow it in the present study.
- We indicated that we would report *years practicing* as *age began practicing* subtracted from *mean age* (our sample's mean and Ericsson et al.'s sample's mean) because this is how Ericsson et al. describe years practicing. However, this inflates the number of years practicing for students younger than the mean. We thus chose to report the more accurate measure of *years practicing* = *age began practicing* subtracted from *current age*. Information for all calculations is included in the Open Data.
- We did not conduct a *t*-test comparing growth mindsets for those familiar with deliberate practice and/or the 10,000-hour rule because only 3 participants were not familiar with either.
- We did not conduct independent *t*-tests comparing the professional violinists to the best violinists because of the small number of professional violinists. Instead, we report descriptive statistics and graph the data in the Supplemental Materials.
- We replaced standard deviations with 95% confidence interval lower and upper bounds.

References

1. Ericsson, K. A., Krampe, R. T., & Tesch-Römer, C. (1993). The role of deliberate practice in the acquisition of expert performance. *Psychological Review*, 100, 363–406. doi: 10.1037/0033-295X.100.3.363
2. Grammaphone (2012). Retrieved from <https://www.gramophone.co.uk/editorial/the-world%E2%80%99s-greatest-orchestras>
3. Dweck, C. S. (2000). *Self-theories: Their role in motivation, personality, and development.* New York: Psychology Press.
4. Foster, J. L., Shipstead, Z., Harrison, T. L., Hicks, K. L., Redick, T. S., & Engle, R. W. (2015). Shortened complex span tasks can reliably measure working memory capacity. *Memory & Cognition, 43*, 226-236. doi: 10.3758/s13421-014-0461-7
5. Oswald, F. L., McAbee, S. T., Redick, T. S., & Hambrick, D. Z. (2015). The development of a short domain-general measure of working memory capacity. *Behavior Research Methods, 47*, 1343-1355. doi: 10.3758/s13428-014-0543-2
6. Emery, L., Myerson, J., & Hale, S. (2007). Age differences in item manipulation span: The case of letter-number sequencing. *Psychology and Aging, 22*, 75-83. doi: 10.1037/0882-7974.22.1.75
7. Raven, J. C. (1990). *Advanced progressive matrices: sets I, II*. Oxford: Oxford Psychologists Press.
8. Ekstrom, R. B., French, J. W., Harman, H. H., & Dermen, D. (1976). *Kit of factor-referenced cognitive tests.* Educational Testing Service. Princeton, NJ.
9. Wonderlic Personnel Test, Inc. (1992). *Wonderlic Personnel Test and Scholastic Level Exam: User’s manual*. Libertyville, IL: Author.
10. Hicks, K. L., Harrison, T. L., & Engle, R. W. (2015). Wonderlic, working memory capacity, and fluid intelligence. *Intelligence, 50*, 186-195. doi: 10.1016/j.intell.2015.03.005
11. Salthouse, T. A., & Babcock, R. L. (1991). Decomposing adult age differences in working memory. *Developmental Psychology, 27*, 763-776.
12. Ullén, F., Mosing, M. A., Holm, L., Eriksson, H., & Madison, G. (2014). Psychometric properties and heritability of a new online test for musicality, the Swedish Musical Discrimination Test. Personality and Individual Differences, 63, 87-93. doi: 10.1016/j.paid.2014.01.057

1. The lower bound of the 95% confidence interval is calculated as a negative number. Given that it is not possible to have negative minutes of music memorized, we report the lower bound as 0.00. [↑](#footnote-ref-1)
